# Long non-coding RNA MEG3 promotes cisplatin-induced nephrotoxicity through regulating AKT/TSC/mTOR-mediated autophagy

**DOI:** 10.7150/ijbs.58910

**Published:** 2021-09-21

**Authors:** Xu Jing, Jinming Han, Junhao Zhang, Yi Chen, Juan Yuan, Jue Wang, Shiyong Neo, Shuijie Li, Xueyuan Yu, Jing Wu

**Affiliations:** 1 Department of Clinical Laboratory, The Second Hospital of Shandong University, Jinan, 250000, China.; 2 Department of Clinical Neuroscience, Karolinska Institutet, S-171 76, Sweden.; 3 Department of Oncology, Nanfang Hospital, Southern Medical University, Guangzhou, 510515, China.; 4 Department of Oncology-Pathology, Karolinska Institutet, Stockholm, Sweden.; 5 Department of Cell and Molecular Biology, Karolinska Institutet, S-171 76, Sweden.; 6 Key Laboratory, The Second Hospital of Shandong University, Jinan, 250000, China.; 7 Department of Microbiology, Tumor and Cell Biology, Karolinska Institutet, S-171 76, Sweden.; 8 Department of Nephrology, Qilu hospital of Shandong University, Jinan, China.; 9 Department of Pharmacology, The Second Hospital of Shandong University, Jinan, 250000, China.

**Keywords:** *lncRNA MEG3*, DDPIN, miRNA-126, autophagy

## Abstract

Cis-Diamminedichloroplatinum (II) (DDP)-induced nephrotoxicity (DDPIN) may cause irreversible renal injury associated with high morbidity and mortality. Current standard therapies have not achieved satisfactory clinical outcomes due to unclear molecular and cellular mechanisms. Therefore, exploring potential therapies on DDPIN represents an urgent medical need. Present study characterized the role of lncRNA maternally expressed gene 3 (*lnc-MEG3*) in the pathogenesis of DDPIN. In both *in vitro* and in murine models of DDP-induced nephrotoxicity, *lnc-MEG3* exacerbated DDPIN by negatively regulating miRNA-126 subsequently causing a decreased AKT/TSC/mTOR-mediated autophagy. By silencing *lnc-MEG3* or incorporating miRNA-126 mimetics, the proliferation and migration of DDP-treated cells were restored. *In vivo*, we identified Paeonol to alleviate DDPIN by the inhibition of *lnc-MEG3*. Taken together, *lnc-MEG3* represents a novel therapeutic target for DDPIN and Paeonol may serve as a promising treatment by inhibiting *lnc-MEG3* and its related signaling pathways.

## Introduction

Cisplatin (cis-diamminedichloroplatinum (II), DDP) is a widely used chemotherapy for the treatment of many malignancies 1-4. However, DDP-induced nephrotoxicity (DDPIN) may cause irreversible renal injury in a dose-dependent manner, causing discontinuation of this therapy 5, 6. Prophylactic approaches, aimed at offsetting these adverse effects, include hydration therapy and anti-oxidants (e.g., glutathione). However, these approaches had limited beneficial long-term effects 7, 8. Precise cellular and molecular mechanisms of DDPIN are unclear, representing a significant barrier for developing effective therapies that can be given alongside DDP.

Long non-coding RNAs (lncRNAs), molecules longer than 200 nucleotides, directly or indirectly interact with target genes at the transcriptional level and play an important role in biological processes 9. Recent data suggested that lncRNAs play a crucial role in the pathogenesis of renal diseases including kidney ischemia-reperfusion injury 10. lncRNA maternally expressed gene 3 (*lnc-MEG3*), transcripts 1.6kb, is an imprinted gene locus located at chromosome 14q32.3 in humans and chromosome 12 in mice. Previous studies have demonstrated that *lnc-MEG3* was reduced in renal fibrosis and renal cell carcinoma 11, 12. In addition, the inhibition of *lnc-MEG3* has been shown to significantly alleviate lipopolysaccharide-induced renal tubular epithelial cell apoptosis and hypoxia-elicited kidney injury in acute renal allografts 13-15. However, the role of *lnc-MEG3* in DDPIN has not been established yet.

lncRNAs usually function as a competing endogenous RNA (ceRNA) sponge to regulate microRNA expression and downregulate specific proteins such as AKT 9. AKT is known to phosphorylate TSC2, an important component of the TSC complex, by inhibiting mTOR activation 16. Suppression of mTOR-mediated autophagy resulted in renal injury since basic autophagy plays a cytoprotective role in DDPIN 17.

Paeonol (Pae, 2-hydroxy-4-methoxyacetophenone) is an active ingredient derived from the root bark of the Chinese herbal medicine* Moutan Cortex* 18. Pae has several biologic functions including anti-oxidant and anti-inflammatory effects 19-22. We have previously indicated that Pae exerts protective effects on chemotherapeutic drugs-induced toxicities 23-26.

In this study elevated level of *lnc-MEG3* in DDPIN was noted both *in vitro* and *in vivo* and subsequently suppressed the activation of miRNA-126. Inhibition of miRNA-126 activated the AKT/TSC pathway, leading to suppressed mTOR-mediated autophagy, inflammatory responses, apoptosis and renal tubular epithelial cell damage. We further uncover a novel mechanism in which Pae exhibits protective effects on DDPIN by inhibiting the ability of *lnc-MEG3* to regulate miRNA-126.

## Results

### *lnc-MEG3* is upregulated significantly in DDPIN and lnc-MEG3 silencing alleviates DDP-induced inflammation and apoptosis

**Figure 1:**
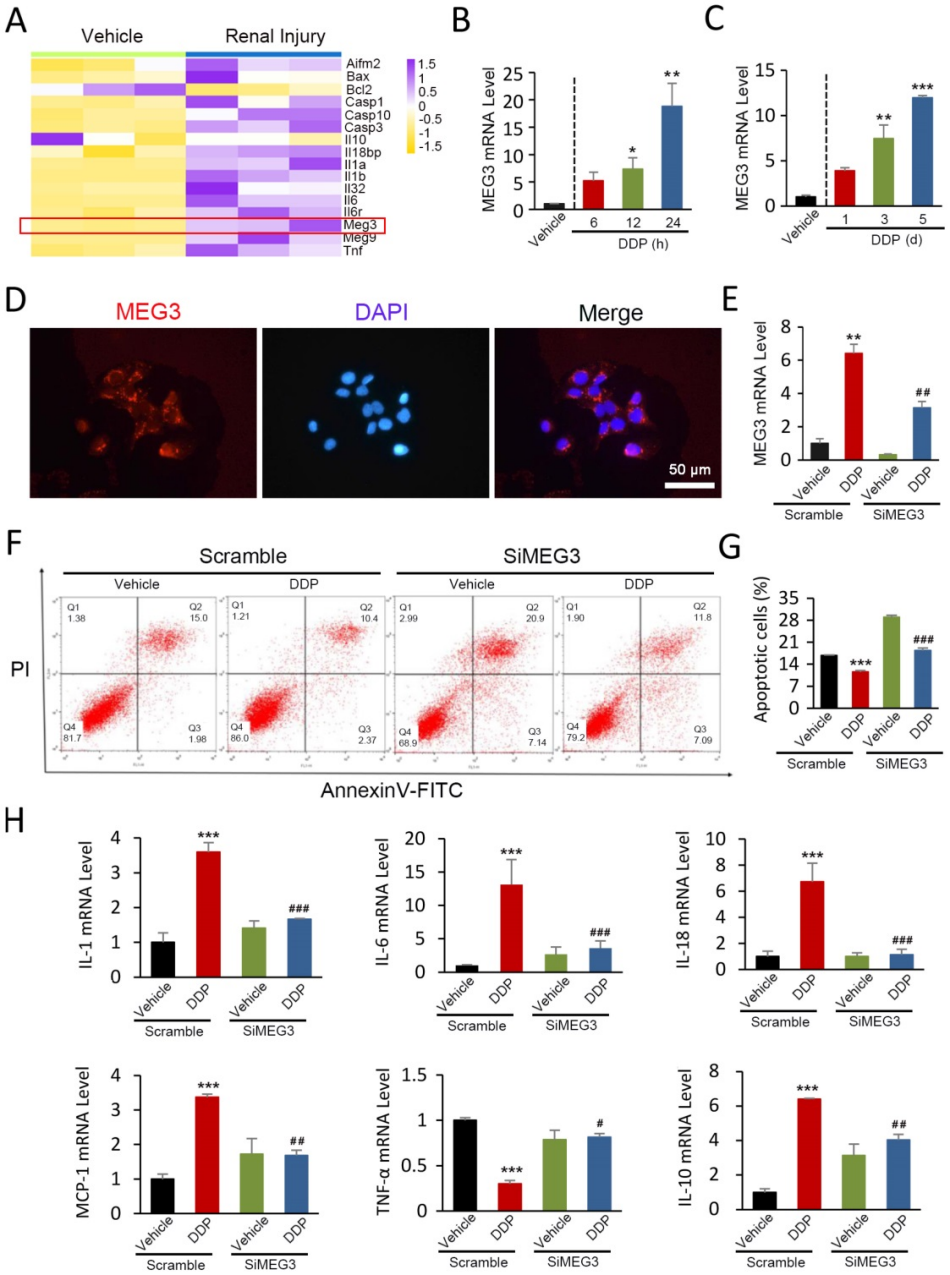
***lnc-MEG3* is upregulated significantly and *lnc-MEG3* silencing alleviates DDP-induced inflammation and apoptosis. (A)** *lnc-MEG3* expressions were visualized by R package “pheatmap” based on the RNA sequence from GEO database. Relative mRNA levels of *lnc-MEG3* in DDP-treated HK-2 cells (**B**), the kidneys of DDPIN mice (**C**) on RT-PCR analysis. (**D**) FISH assays of *lnc-MEG3* location at the cytoplasm fractionation of proximal tubule epithelial cells. Relative mRNA levels of *lnc-MEG3* silenced HK-2 cells on RT-PCR analysis (**E**). Flow cytometry (**F**) and data analysis (**G**) of apoptosis in DDPIN or/and *lnc-MEG3* silenced HK-2 cells. **(H)** RT-PCR analysis of IL-1, IL-6, IL-10, IL-18, MCP-1 and TNF-α. *P < 0.05, **P < 0.01, ***P < 0.001 versus control, ^#^*P* < 0.05, ^##^*P* < 0.01, ^###^*P* < 0.001 versus DDP-treated control (n=3).

By first looking into the differentially expressed genes during renal injury, we identified that the expression of *lnc-MEG3* was drastically in renal injury based on publicly available NCBI Gene Expression Omnibus (GEO) database (GSE124622) (Figure 1A). To investigate the role of *lnc-MEG3* in DDP-induced renal injury and dysfunction, 30 mg/kg DDP was injected into the mice to establish a DDPIN mice model. Increased serum levels of urea nitrogen (BUN) and creatinine (SCr) (Figure S1A); severe morphological changes including loss of the tubule dilatation and cast formation (Figure S1B); increased cell death (Figure S1C); accumulation of neutrophils and CD68^+^ macrophages (Figure S1D) and alteration of inflammatory cytokines (Figure S1E) were evident, highlighting the validity of our DDPIN mice model. Consistently, the expression of *lnc-MEG3* was dramatically elevated in a time-dependent manner in both DDP-treated HK-2 cells and DDPIN mice (Figure 1B and 1C).

Fluorescence *in situ* hybridization (FISH) assays were used to examine the location and expression of *lnc-MEG3* mRNA and our results demonstrated that *lnc-MEG3* localized primarily in the cytoplasm fractionation of proximal tubule epithelial HK-2 cells (Figure 1D). We then used siRNA to knock down *lnc-MEG3* in HK-2 cells (Figure 1E and Figure S2A). Our findings showed that *lnc-MEG3* silencing significantly decreased DDP-induced apoptosis of HK-2 cells, as measured by MTT (Figure S2B) and flow cytometry (Figure 1F and 1G). The expression of increased proinflammatory cytokines and decreased anti-inflammatory factors in the condition of DDP was reversed after *lnc-MEG3* silencing (Figure 1H). Collectively, *lnc-MEG3* plays a vital role in DDP-induced inflammation and apoptosis and *lnc-MEG3* silencing exerts beneficial effects, alleviating DDPIN.

### AKT/TSC/mTOR-mediated autophagy is impaired in DDPIN

**Figure 2:**
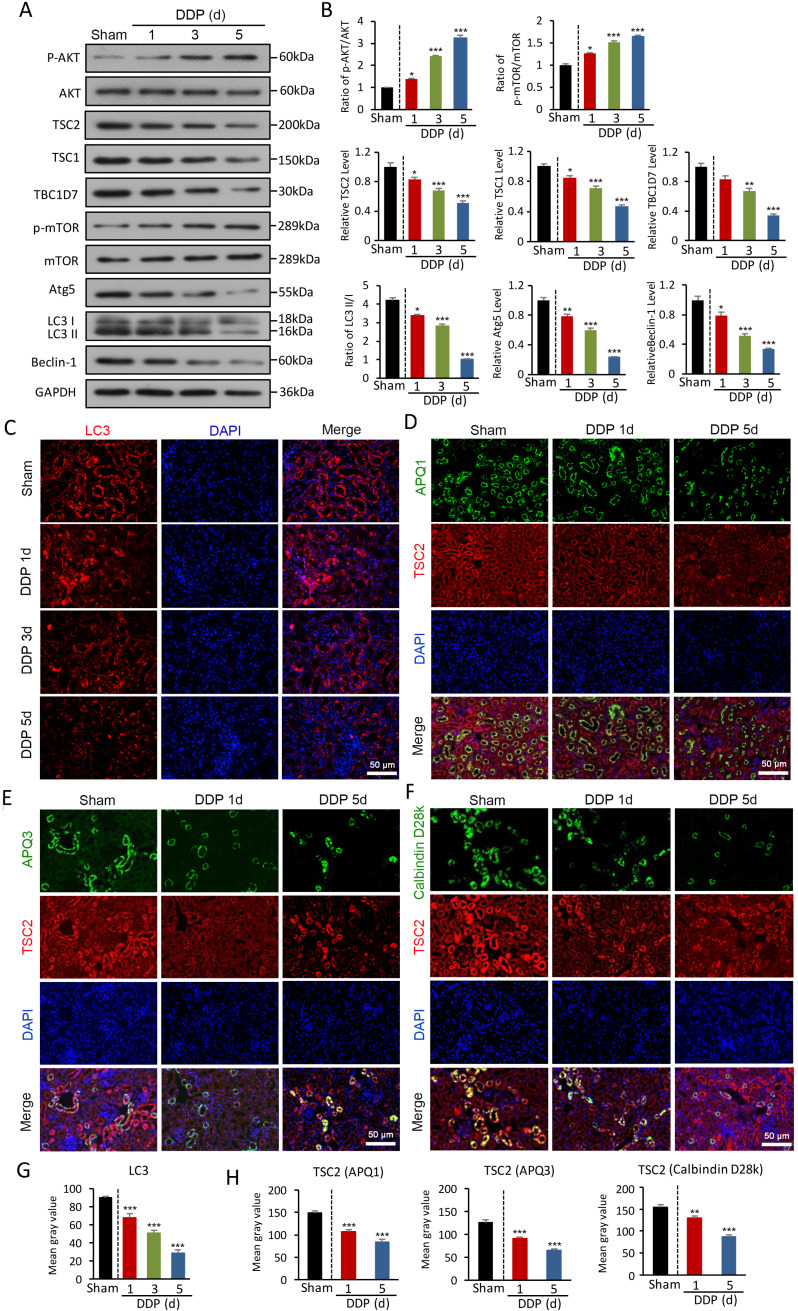
** AKT/TSC/mTOR-mediated autophagy is impaired in DDPIN. (A and B)** Western blot analysis of p-AKT, AKT, TSC2, TSC1, TBC1D7, p- mTOR, mTOR, Atg5, Beclin-1 and LC3 in the kidneys of DDPIN mice. **(C)** Representative images showing LC3 staining in the kidneys of DDPIN mice. **(D-F)** Co-immunofluorescence staining for TSC2 and tubular segment-specific markers in the kidney of DDPIN. The following segment-specific tubular markers were used: proximal tubule, aquaporin-1 (AQP1); distal tubule, calbindin D28k; and collecting duct, aquaporin-3 (AQP3). **(G-H)** The data analysis of LC3 and TSC2. *P < 0.05, **P < 0.01, ***P < 0.001 versus control (n=3).

Given impaired autophagy results in apoptosis and inflammation in DDPIN, we sought to define relevant autophagy signaling pathways in DDPIN. We first found that AKT was highly phosphorylated upon DDP stimulation in a dose-dependent manner (Figure 2A and 2B). Consequently, the activation of the tuberous sclerosis complex (TSC) including TSC1, TSC2, and TBC1D7 was inhibited. Furthermore, the suppression of TSC2 promoted mTOR phosphorylation, which led to a decline in autophagy-related molecules (Figure 2A and 2B). Consistently, we observed reduced LC3 immunofluorescence staining in the kidney of DDPIN mice (Figure 2C). We then looked for TSC2 and various tubular markers in kidney tissues to determine if TSC2 can be scaffolded to TSC1 and TBC1D7 to maintain the stability of the TSC complex and inhibit mTOR signaling. To identify TSC2 localisation within the kidney, we costained aquaporin-1 (AQP1), Calbindin (D28K) and aquaporin-3 (AQP3). As shown in Figure 2D and 2F, TSC2 could be found in proximal tubules (AQP1+ cells), distal tubules (D28K+ and AQP3+ cells) . From image analysis, we indeed confirmed that LC3 and TSC2 were downregulated upon DDP treatment (Figure 2G and 2H). Indeed, our DDPIN model revealed impaired autophagy in multiple tissue sites of the kidney and from here, we sought to find out if these cellular dysfunction is related to lnc-MEG3.

### *lnc-MEG3* silencing reverses DDP-induced defects in AKT/TSC/mTOR-mediated autophagy

**Figure 3:**
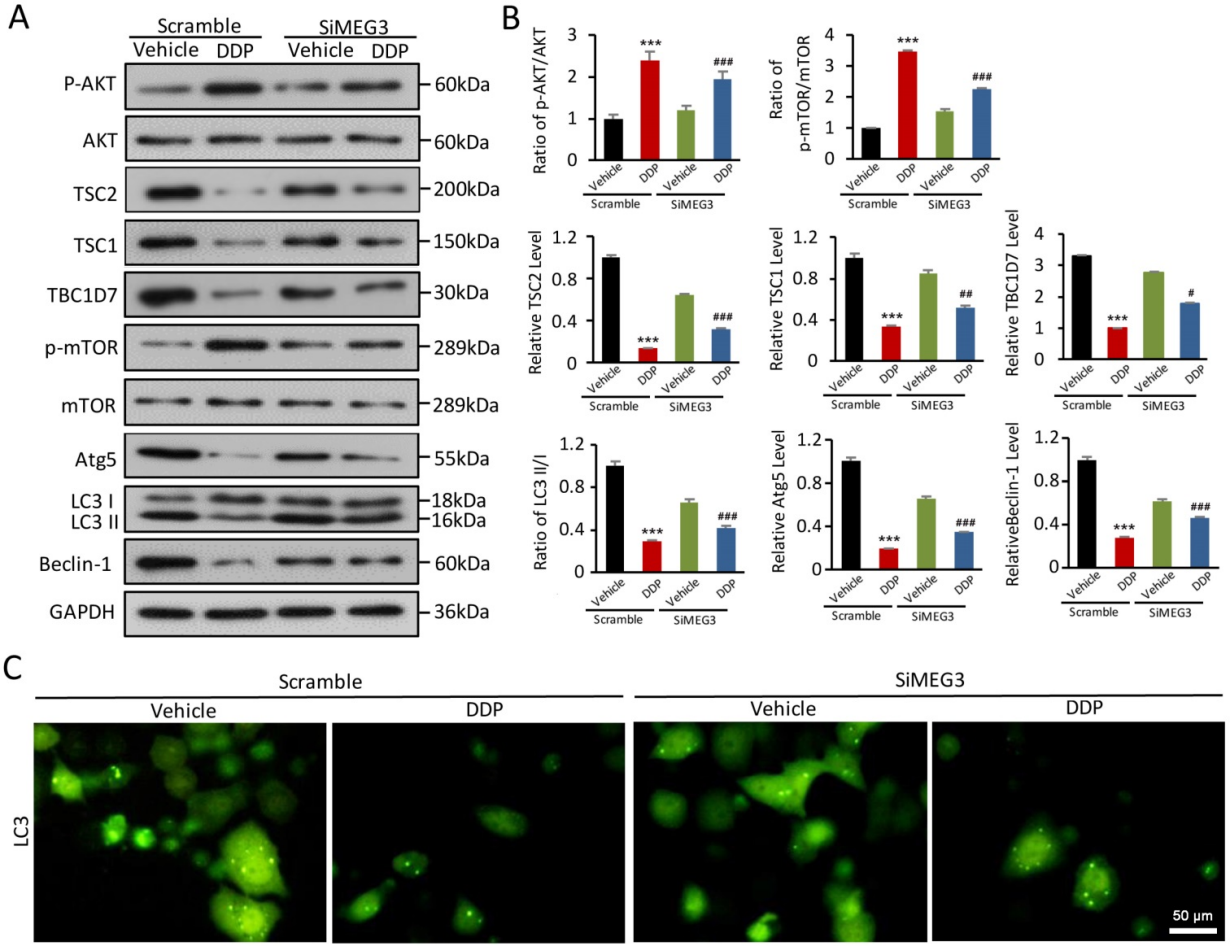
***lnc-MEG3* silencing reverse the impaired AKT/TSC/mTOR-mediated autophagy induced by DDP. (A and B)** Western blot analysis of p-AKT, AKT, TSC2, TSC1, TBC1D7, p-mTOR, mTOR, Atg5, Beclin-1 and LC3 in the DDP-treated and/or *lnc-MEG3* silenced HK-2 cells. **(C)** Immunofluoresence of LC3 staining in the DDP-treated HK-2 cells under the condition of *lnc-MEG3* silence. *P < 0.05, **P < 0.01, ***P < 0.001 versus control, ^#^*P* < 0.05, ^##^*P* < 0.01, ^###^*P* < 0.001 versus DDP-treated control (n=3).

A plausible hypothesis to investigate was if *lnc-MEG3* contributes to DDP-induced autophagy dysfunction. Using Western blot, we found that the increased AKT phosphorylation induced by DDP could be reversed by *lnc-MEG3* silencing (Figure 3A and 3B). Notably, TSC complex components TSC1, TSC2, and TBC1D7 were elevated following *lnc-MEG3* silencing and TSC2 is a known downstream targeted molecule of mTOR (Figure 3A and 3B). Furthermore, the expression of autophagy-related proteins including LC3, Atg5 and Beclin-1 were increased following *lnc-MEG3* silencing (Figure 3A and 3B) and an increased expression of LC3 was also observed (Figure 3C). These findings suggest that *lnc-MEG3* can be targeted to restore AKT/TSC/mTOR-mediated autophagy in DDPIN.

### miRNA-126 is negatively regulated by* lnc-MEG3* and ablates DDP-induced autophagy dysfunction

**Figure 4:**
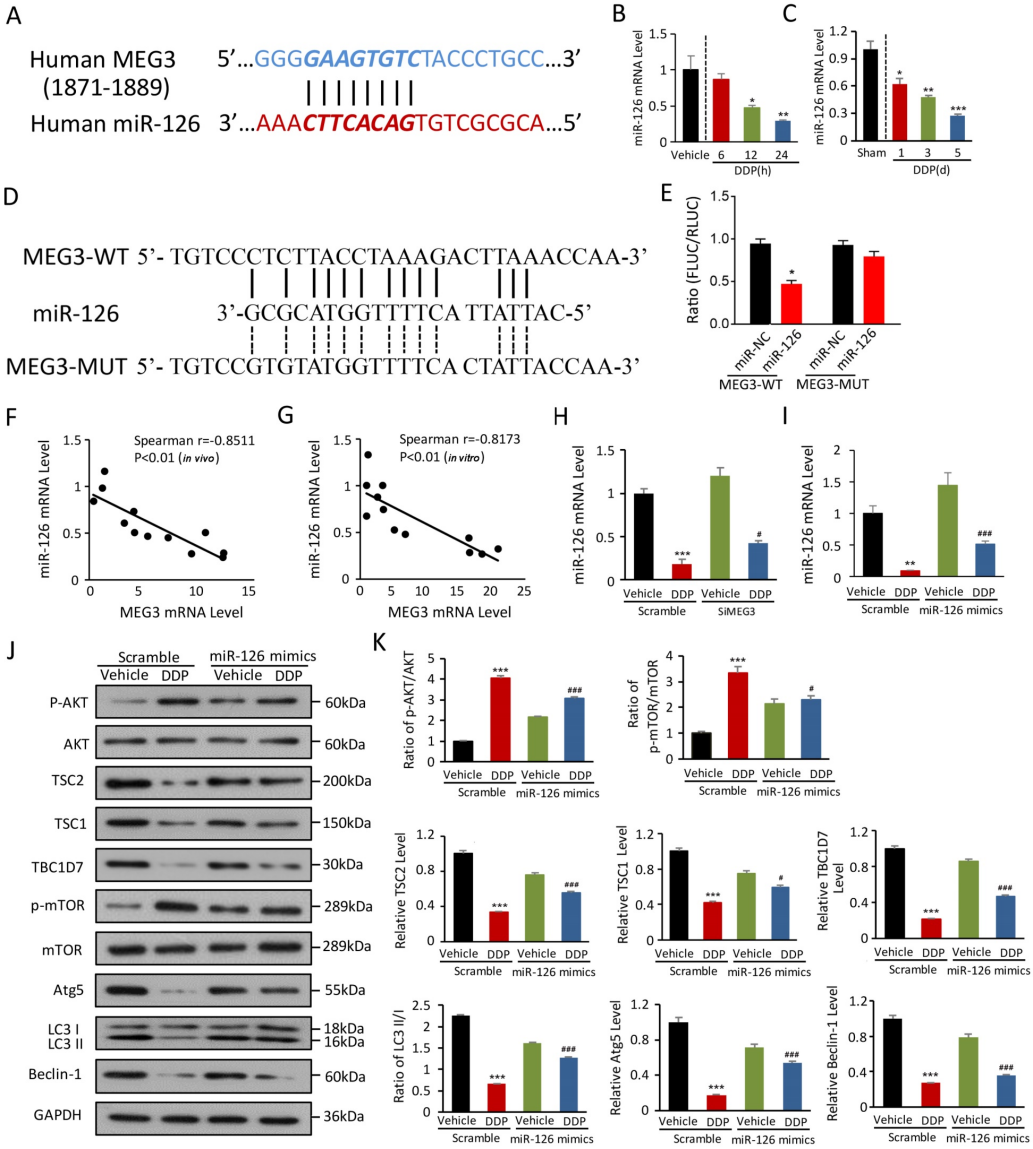
** miRNA-126 is negatively regulated by *lnc-MEG3* and ablates DDP-induced autophagy depression. (A)** The targeting relationship between* lncRNA MEG3* and miRNA-126. Relative mRNA level of miRNA-126 in HK-2 cells (**B**) and the kidney of DDPIN mice (**C**). The luciferase activity in the MEG3-WT and MEG3-MUT compared to control group, and the elements responsible for miR126 expression are between -1871 and -1889 as shown in (D) and (E). Negative correlation between *lnc-MEG3* and miRNA-126 *in vivo* (**F**) and *in vitro* (**G**). Relative mRNA level of miRNA-126 under the condition of *lnc-MEG3* silencing or miRNA-126 mimics in DDP-treated HK-2 cells, respectively (**H and I**). **(J and K)** Western blot analysis of p-AKT, AKT, TSC2, TSC1, TBC1D7, p-mTOR, mTOR, Atg5, Beclin-1 and LC3 in the DDP-treated and/or miRNA-126 mimics HK-2 cells. *P < 0.05, **P < 0.01, ***P < 0.001 versus control, ^#^*P* < 0.05, ^##^*P* < 0.01, ^###^*P* < 0.001 versus DDP-treated control (n=3).

lncRNAs can function as a competing endogenous RNA to sponge miRNAs, indicating how lnc-MEG3 exerts suppressive effects on autophagy regulation. *In silico* analysis predicted miRNA-126 as a binding of *lnc-MEG3* (Figure 4A). miRNA-126 expression was dramatically reduced in the kidney of DDPIN mice and DDP-treated HK-2 cells (Figure 4B and 4C). To determine if MEG3 is responsible for miRNA-126 expression, various luciferase reporters with MEG3-WT and MEG3-MUT were constructed and transiently transfected into HK2 cells. Our results showed that the MEG3-WT fragment contains the elements required for miRNA-126 expression in the MEG3-WT group. However, the luciferase activity was ablated in the MEG3-MUT group, indicating that the elements responsible for miRNA-126 expression are noted between -1871 and -1889 as shown in Figure 4D and 4E. The level of miRNA-126 was also inversely correlated with *lnc-MEG3* expression both *in vivo* (Spearman r = -0.8511, *P<0.01*; Figure 4F) and *in vitro* (Spearman r = -0.8173, *P<0.01*; Figure 4G). Furthermore, miRNA-126 and miRNA-126 mimics expression were elevated in *lnc-MEG3* knock-down HK-2 cells (Figure 4H and 4I).

To explore additional functions of miRNA-126 in DDPIN, we used MTT to evaluate the impact of miRNA-126 on proliferation. We found that the overexpression of miRNA-126 could increase the inhibition of proliferation caused by DDP in HK-2 cells (Figure S2C), indicating that miRNA-126 plays a protective role in DDPIN. We then evaluated protein expression in the AKT/TSC/mTOR-mediated autophagy signaling pathway (Figure 4J and 4K). We also found that miRNA-126 impaired DDP-induced autophagy dysfunction, consistent with the function of si-*lnc-MEG3* (Figure 4J and 4K). Collectively, these results suggested that miRNA-126 is negatively regulated by *lnc-MEG3*, implicating critical cellular functions such as autophagy and proliferation.

### *lnc-MEG3* silencing and miRNA-126 mimics are beneficial for the proliferation and migration in DDP-treated HK-2 cells

**Figure 5:**
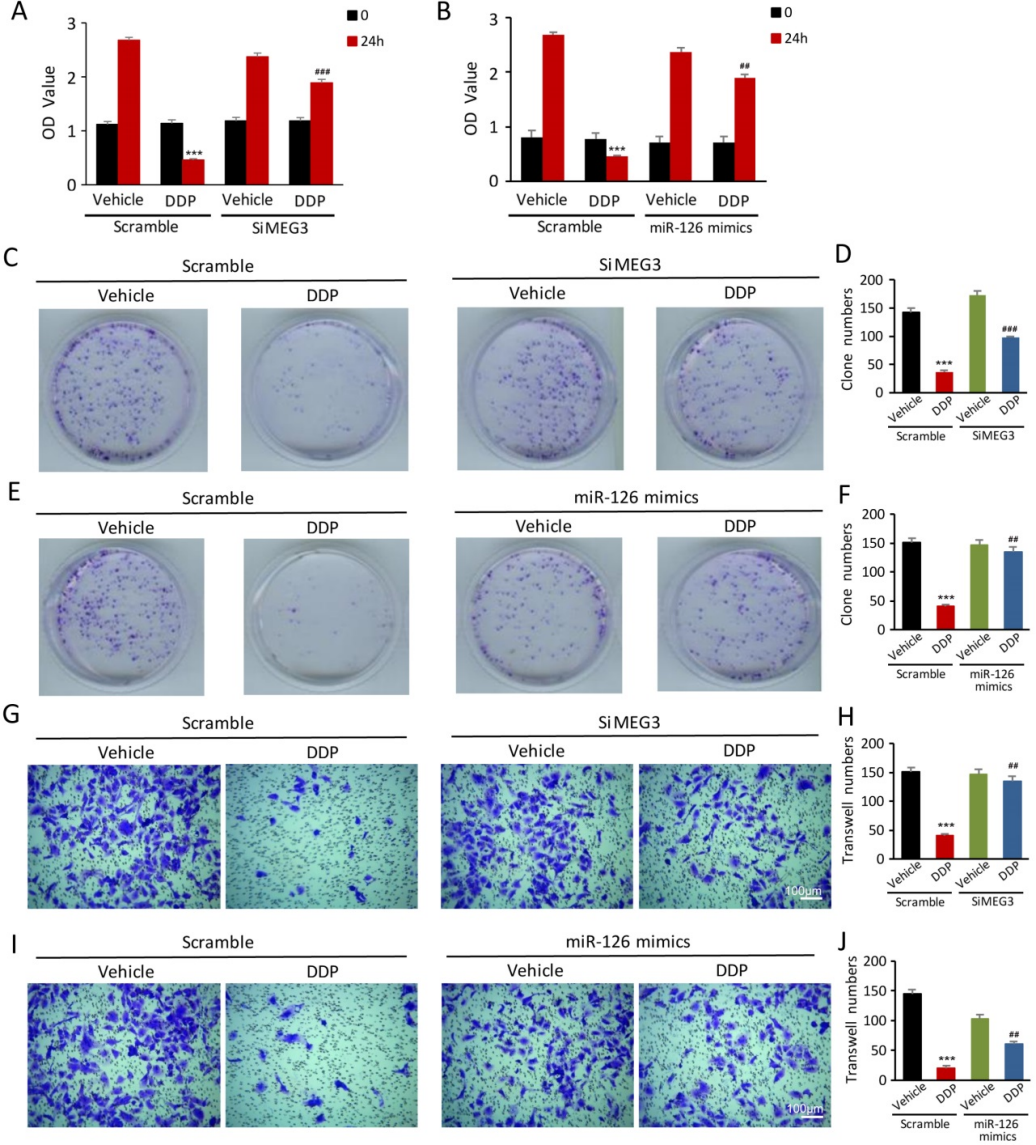
***lnc-MEG3* silencing and miRNA-126 mimics are beneficial to the proliferation and migration of the DDP-treated HK-2 cells. (A and B)** CCK8 assay, **(C-F)** Colony formation assay, Transwell assay of HK-2 cell (**G-J**) in the condition of *lnc-MEG3* silence or miRNA-126 mimics. *P < 0.05, **P < 0.01, ***P < 0.001 versus control, ^#^*P* < 0.05, ^##^*P* < 0.01, ^###^*P* < 0.001 versus DDP-treated control (n=3).

To validate if *lnc-MEG3* and miRNA-126 can affect the proliferation and migration of DDP-treated HK-2 cells, the CCK8 assay, colony formation, and transwell assays methods were used. The proliferation of HK-2 cells was inhibited following DDP treatment, as measured by the CCK8 assay. Strikingly, *lnc-MEG3* silencing and miRNA-126 mimetics restored the suppressed proliferation of HK-2 cells induced by DDP (Figure 5A and 5B). Likewise, DDP treatment reduced colony formation. Knockdown of *lnc-MEG3* (Figure 5C and 5D) and overexpression of miRNA-126 (Figure 5E and 5F) restored colony formation function of proximal tubule cells following DDP treatment. Additionally, we found that a significant reduction of cell migration after DDP treatment could be reversible by *lnc-MEG3* silencing (Figure 5G and 5H) and miRNA-126 overexpression (Figure 5I and 5J). MiRNA-126 inhibitor was also found to downregulate the proliferation and migration of the HK-2 cells significantly (Figure S3).

### Pae protects against DDPIN via regulation of *lnc-MEG3*/miRNA-126/mTOR-mediated autophagy

**Figure 6:**
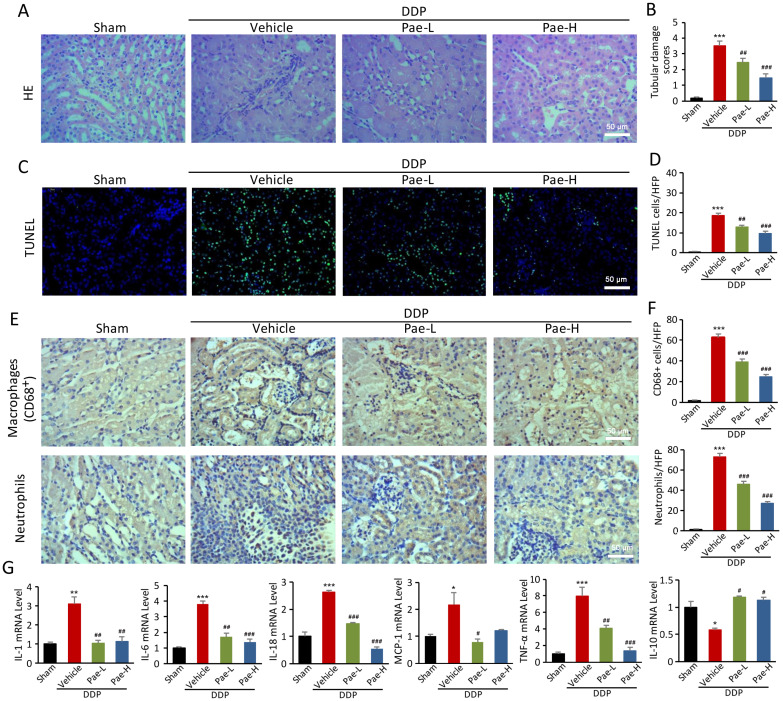
Pae functions as an effective protection in DDPIN. HE staining, (**A and B**) TUNEL staining, (**C and D**), Immunohistochemical staining of neutrophils and CD68+ macrophages (**E and F**) and RT-PCR analysis (**G**) of IL-1, IL-6, IL-10, IL-18, MCP-1 and TNF-α in DDP or/and Pae-treated (15 mg/kg and 30 mg/kg) mice. *P < 0.05, **P < 0.01, ***P < 0.001 versus control, ^#^*P* < 0.05, ^##^*P* < 0.01, ^###^*P* < 0.001 versus DDP-treated control (n=3).

We have previously reported that Pae can protect against chemotherapy-induced renal injury in mice 25. Pae alleviates DDPIN at the DDP doses of 15 and 30 mg/kg. Specifically, BUN and SCr values normalized in DDP-treated mice following Pae administration, compared to the control mice (Figure S4). Other features of DDPIN including morphological injury (Figure 6A and 6B), apoptosis (Figure 6C and 6D), accumulation of neutrophils and CD68^+^ macrophages (Figure 6E and 6F) and proinflammatory inflammatory factors (Figure 6G) improved dramatically after Pae treatment. In addition, Pae could reverse DDP-induced apoptosis, decrease clonogenic and migration ability *in vitro (*Figure S5).

**Figure 7:**
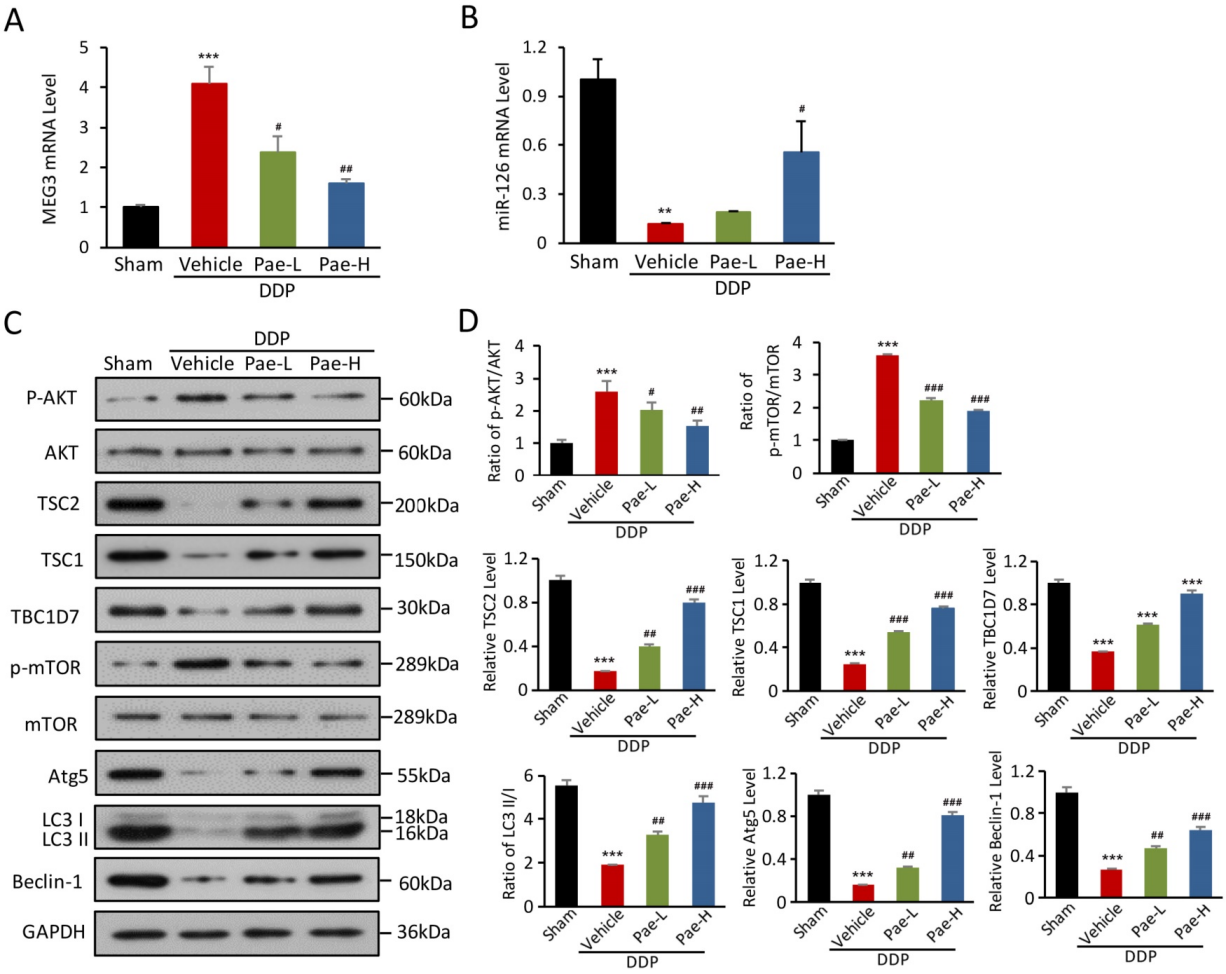
** Pae protects against DDPIN through regulating *lnc-MEG3* /miRNA-126/mTOR-mediated autophagy.** Relative mRNA level of *lnc-MEG3* (**A**) and (**B**) miRNA-126 in DDP or/and Pae-treated (15 mg/kg and 30 mg/kg) mice. **(C and D)** Western blot analysis of p-AKT, AKT, TSC2, TSC1, TBC1D7, p-mTOR, mTOR, Atg5, Beclin-1 and LC3 in the DDP or/and Pae-treated (15 mg/kg and 30 mg/kg) mice. *P < 0.05, **P < 0.01, ***P < 0.001 versus control, ^#^*P* < 0.05, ^##^*P* < 0.01, ^###^*P* < 0.001 versus DDP-treated control (n=3).

To explore protective mechanisms of Pae in DDPIN, we evaluated the expression of *lnc-MEG3* and miRNA-126 in DDPIN mice. Pae effectively alleviated DDP-induced *lnc-MEG3* elevation and miRNA-126 reduction (Figure 7A and 7B). We also found that Pae affected AKT/TSC/mTOR axis-mediated autophagy (Figure 7C and 7D). In addition, Pae could decrease the *lnc-MEG3* expression (Figure S6A), increase miRNA-126 level (Figure S6B) and affect inflammatory factors (Figure S7). Evidently, these findings represented a novel mechanism underlying the protective effects of Pae in mice with DDPIN.

## Discussion

Nephrotoxicity is a severe complication that limits clinical use of cisplatin in cancer therapy, with at least one-third of patients treated with cisplatin suffering from renal injury 27. A variety of cellular and molecular mechanisms have been proposed for cisplatin-induced renal injury including ROS production 28, DNA damage 29, P53 activation 30 and cell cycle alteration 31. Although multiple renal protective approaches have been established, current therapies do not achieve a satisfactory outcome.

Autophagy is considered as a double-edged sword at physiological levels, serving as a critical safeguard against kidney dysfunction. 32, 33. Emerging data has shown that DDP augments autophagy and triggers apoptosis and inflammation 17. However, it has also been reported that autophagy plays a cytoprotective role in cisplatin-induced AKI 34. One explanation for these conflicting results is that diverse stimuli and environments are responsible for differing stages of autophagy. Nonetheless, autophagy plays a protective role in inflammatory disorders by adjusting interferon production or inflammasome activation 14. We have previously demonstrated that autophagy inhibition promotes podocyte injury in diabetic nephropathy 35. In this study, we found that autophagy levels are significantly decreased in DDPIN mice and HK-2 cells, which is accompanied by severe apoptosis and inflammation. In response to toxic side effects of drugs, autophagy is enhanced to prevent the kidney from tubular damage and its consequent injury 36. Previous reports have shown that autophagy protects kidney proximal tubules from acute injury by reducing DNA damage and decreasing ROS production 36. Consistent with these results, we observed that autophagy upregulation could alleviate the severe apoptosis observed in DDPIN.

We then explored how cisplatin regulates autophagy levels and subsequent renal injury. We found that *lnc-MEG3* was significantly increased in DDPIN. *lnc-MEG3* is expressed in several human normal tissues, yet is notably absent in various types of human carcinomas 37-39, suggesting it may act as a tumor suppressor. Previous studies have shown that it can inhibit tumor cell proliferation and induce apoptosis by stabilizing and activating p53 40. More recently, researchers have indicated that *lnc-MEG3* was a vital regulator to promote the impairment of diastolic function 38 and knockdown of *lnc-MEG3* alleviates hypoxia induced cardiomyocyte injury 41. *lnc-MEG3* expression has been closely tied to many kidney diseases including transforming growth factor β1 (TGF-β1)-induced renal fibrosis 11, LPS-induced renal tubular epithelial cells apoptosis 42 and hypoxia-induced kidney injury 43. Despite the known association between *lnc-MEG3* and kidney injury, studies of *lnc-MEG3* in the context of DDPIN are lacking. It was reported that *lnc-MEG3* knockdown alleviates apoptosis or inflammation in human bronchial epithelial cells and macrophages 44. In line with above findings, we observed that *lnc-MEG3* was increased in DDPIN, which was associated with increased apoptosis and inflammation. These can be reversible following silencing *lnc-MEG3*. Expression of autophagy-related proteins and autophagy-associated AKT/TSC/mTOR signaling was also significantly increased upon *lnc-MEG3* knock down and *lnc-MEG3* silencing. Collectively, our results suggest that *lnc-MEG3* functions as a critical mediator of DDPIN by regulating AKT/TSC/mTOR-mediated autophagy.

We then studied how *lnc-MEG3* regulates autophagy-related signaling pathways based on a bioinformatic prediction that *lnc-MEG3* may function as a competing endogenous RNA sponge to regulate the miRNA-126 expression. In fact, miRNA-126 was negatively correlated with *lnc-MEG3* both *in vitro* and *in vivo* and the downregulation of miRNA-126 expression was observed in DDPIN, whereas miRNA-126 overexpression reversed characteristic features of DDPIN. To clarify the downstream signaling pathways mediated by miRNA-126 we analyzed the activation of intracellular autophagy signaling components. As expected, AKT was highly phosphorylated in DDPIN mice and was accompanied by increased *lnc-MEG3* and decreased miRNA-126 expression. AKT is known to phosphorylate TSC2, which destabilizes the TSC2 protein and results in TSC complex (i.e., TSC1, TCS2 and TBC1D7) dissociation from the lysosome 45. The TSC complex inhibits mTOR activation and is driven by increased levels of GTPase-activating protein (GAP)-bound Rheb 46. Thus, DDP-induced elevated *lnc-MEG3* negatively regulates miRNA-126 expression which leads to the activation of AKT/TSC/mTOR signaling pathway that is required for autophagy suppression.

We have previously found that Pae, an active component isolated from *Mudan Cortex*, has protective effects on chemotherapy drugs-induced oxidative stress, nitrative stress and autophagy in the heart, liver and kidney 23-26. Here, we show that Pae reduces the deleterious effects of DDPIN through a reduction in apoptosis and inflammation, suggesting it has a renoprotective effect. Specifically, elevated *lnc-MEG3* and reduced miRNA-126 were reversed in DDPIN mice following Pae treatment. Pae also altered multiple aspects of the AKT/TSC/mTOR signaling pathway, thereby partially ameliorating pathogenic levels of autophagy. Nonetheless, the underlying mechanism in which Pae downregulates lnc-MEG3 remained elusive. Future studies should also look into autopsy specimens of patients who suffered from cisplatin-induced nephropathy. In doing so, the clinical relevance of lnc-MEG3 expression could be further highlighted, inspiring novel therapeutic interventions to alleviate the disease.

To conclude, present study found that *lnc-MEG3* contributed to DDPIN pathogenesis both *in vitro* and *in vivo*. Mechanistically,* lnc-MEG3* functions as an endogenous miRNA-126 sponge which inhibits AKT/TSC/mTOR-mediated autophagy. Impaired autophagy in HK-2 cells and kidney tissues of DDPIN mice then leads to increased apoptosis and inflammation. We also demonstrated that Pae could inhibit the expression of *lnc-MEG3* to attenuate renal injury in DDPIN mice. Our study defines a novel role of *lnc-MEG3*-mediated DDPIN progression and identifies Pae as a promising therapeutic agent in cisplatin-induced nephrotoxicity.

## Materials and methods

### Ethics statement

This study was approved by the Medical Ethics Committee of the Shandong University Medical School (Shandong, China) and conducted in accordance with the National Institutes of Health Guide for the Care and Use of Laboratory Animals (approval number: KYLL-2017(GJ)A-0028).

### Fluorescent *in situ* hybridization (FISH) assays

*In situ* hybridization was performed using the BOSTER kit (BOSTER, BIO. USA) according to the manufacturer's instructions.

### Mouse models of acute kidney injury

Acute kidney injury (AKI) was induced in male C57BL/6 mice aged 6-8 weeks via a single intraperitoneal injection of cisplatin at a dose of 30 mg/kg (Sigma, St. Louis, MO) 47. Mice were sacrificed at 1 d, 3 d and 5 d post-injection. The treatment groups were given Pae (low dose: 15 mg/kg and high dose: 30 mg/kg) by gavage for three consecutive days following cisplatin injection 23.

### Assessment of renal function

The protocol was performed as described previously 48. Blood samples of the mice were collected into a centrifuge and the serum was separated by centrifugation at 1900×g for 5 min.

The Agilent 1100 HPLC system (Agilent Technologies, Santa Clara, CA) was used to measure blood urea nitrogen (BUN), and serum creatinine (SCR).

### Morphological examinations

Kidney sections were fixed using formalin, embedded in paraffin and sliced into 5 μm pieces.The samples were stained with hematoxylin and eosin (H&E). The percentage of tubules in the corticomedullary junction that displayed cellular necrosis and loss of brush border were counted and scored 0-5 in a blinded manner as follows: none, 0-10%, 11-25%, 26-45%, 46-75% and >75%. More than 6 high power fields (200 X) per section for each sample were examined 49.

### Immunohistochemistry

Renal tissues from AKI mice were fixed with formalin, embedded in paraffin and sliced into 5 μm pieces. The slides were stained with different antibodies. (Table S1). Results were analyzed via diaminobenzidine staining (GTVision™ III Detection System Mo&Rb, Gene Tech, Shanghai PR China) under a microscope (Olympus BX60, Tokyo).

### Terminal Deoxynucleotidyl Transferase (TdT)-Mediated 20-Deoxyuridine 50-Triphosphate (dUTP) Nick End Labeling (TUNEL) Staining

Apoptosis in the kidney tissues after DDP (or and Pae) treatment was detected by TUNEL assay, which was performed according to the manufacturer's protocol (Roche Diagnostics, Mannheim, Germany). Apoptotic cells were identified by green nuclear staining counted with one hundred cells from 6 random fields. Results were analyzed via diaminobenzidine staining (GTVision™ III Detection System Mo&Rb, Gene Tech, Shanghai PR China) under a microscope (Olympus BX60, Tokyo).

### Cell culture and treatments

The human tubular epithelial cell line HK-2 was purchased from the Cell Bank of the Chinese Academy of Sciences (Shanghai, China). HK-2 cells were cultured and treated as described previously 50. HK-2 cells were treated with cisplatin (50 μM) for 6 h, 12 h and 24 h, respectively.

### RNA extraction and real time polymerase chain reaction (RT-PCR)

Total RNA was isolated from tissues or HK-2 cells using a TRIzol reagent (Invitrogen, Life Technologies, USA) according to the Manufacturers' instructions as described previously 51. mRNA levels of target genes were measured by real-time RT-PCR (Bio-Rad, Hercules, CA). The primers of target genes are shown in Table S1.

### Flow cytometry

Pre-treated HK-2 cells in different conditions were collected and stained with 7AAD-Annexin V for 30 min. After washed with PBS twice, the samples were detected by a Novocyte flow cytometer (ACEA biosciences). All the data were analyzed by a Flowjo software (BD).

### Western blot analysis

Total protein lysates of kidney tissues or HK-2 cells were prepared for Western blot analysis as described previously 52. Briefly, 20 μL proteins were separated using 12% SDS PAGE gel and electro-transferred to polyvinylidene fluoride membranes (PVDF; Millipore, Billerica, MA, USA).

The membranes were incubated with primary antibodies overnight at 4 °C after blocking. Following incubated with secondary antibodies, the protein bands were visualized by an ECL kit (Millipore) and calculated using the Image Lab software (Bio-Rad, USA). Specific antibodies used are listed in Table S2. GAPDH was used as a loading control.

### Immunofluorescence staining and confocal microscopy

Immunofluorescence staining was performed as described previously 53. A LSM780 laser confocal microscope (ZEISS, Germany) was used to scan the immunofluorescence staining images.

### RNA interference and miRNA mimics

Small interference RNAs (siRNAs) and mimics were synthesized at GeneParma (Shanghai, China) (Table S2). Transfection of siRNA and mimics were performed based on the manufacturer's instructions by using the Bio-Rad siLentFect lipid transfection kit (Bio-Rad, Hercules, CA, USA).

### Cell proliferation assay

HK-2 proliferation was determined by the MTT assay. Briefly, 5×10^3^ HK-2 cells were seeded in a 96-well plate after different treatment conditions. The absorbance of each well was detected at 560 nm.

### CCK8 Assay

CCK8 assay was performed to measure the cell proliferation. After treating different chemicals, cell viability was measured at 0 and 24 h, respectively. Specifically, 10 μL of CCK8 reagent (Solarbio, Beijing, China) was added and cells were incubated at 37 °C for 1 h. The absorbance at 450 nm was measures by a microplate reader.

### Colony Formation Assay

HK-2 cells were fixed with 4% paraformaldehyde for 30 min and stained with 0.1% crystal violet for an additional 30 min following different treatments. Visible colonies were counted in a blinded manner and captured using a microscope (Olympus BX60, Tokyo).

### Transwell Assay

Transwell chambers (8μm pore size; Millipore) were used for cell migration and invasion assays. Specifically, 1×10^5^ cells were plated in the upper chamber and the lower chambers were filled with RPMI 1640 medium containing 10% FBS. Following different treatments, the migrated or invaded cells were fixed with 4% paraformaldehyde for 30 min and stained with 0.1% crystal violet for an additional 20 min.

### Transfection and luciferase assay

The MEG3-WT plasmid was cloned the sequence of MEG3 into the dual luciferase reporter vector pMIR-Glo, MEG3-MUT plasmid was cloned into the pMIR-Glo after mutation of the binding site of MEG3 and miR-126. The miR-126 mimic, MEG3-WT or MEG3-MUT and miR-NC were co-transfected in HK-2 cells using lipofectamine 2000 transfection reagent (Invitrogen) for 48h. Luciferase activities were measured with a Dual Luciferase Reporter Assay System (Promega) according to the manufacturer's instructions. Data are normalized for transfection efficiency by dividing firefly luciferase activity with that of Renilla luciferase.

### Statistical Analysis

Data of mean determinants were presented as ± SEM. No statistical methods were used to predetermine sample sizes, and the exact values of n (sample size) are provided in each figure legends. Statistical analysis of data was performed using a standard Unpaired Student's t-test by GraphPad Prism 8.0. * *P*< 0.05; **P < 0.01; ***P < 0.001; ^#^ *P*< 0.05; ^##^ *P* < 0.01; ^###^ *P* < 0.001; NS, not significant.

## Supplementary Material

Supplementary figures and tables.
