# Supplementary material for: Long non-coding RNA MEG3 promotes cisplatin-induced nephrotoxicity through regulating AKT/TSC/mTOR-mediated autophagy
Source: Int J Biol Sci. 2021 Sep 21;17(14):3968–80. doi: 10.7150/ijbs.58910 (PMC8495387; doi:10.7150/ijbs.58910)
Supplement: Supplementary file 1 — Supplementary figures and tables. [file ijbsv17p3968s1.pdf]

Supporting Information for

Original article

Long noncoding RNA MEG3 promotes cisplatin-induced nephrotoxicity through regulating AKT/TSC/mTOR-mediated autophagy

Xu Jing [1], Jinming Han [2], Junhao Zhang [3], Yi Chen [4], Juan Yuan [5], Jue Wang [6], Shiyong Neo [4], Shuijie Li [7], Xueyuan Yu [8], Jing Wu [9]*

[1] Department of Clinical Laboratory, The Second Hospital of Shandong University, Jinan, 250000, China; [2] Department of Clinical Neuroscience, Karolinska Institute, S-171 76, Sweden; [3] Department of Oncology, Nanfang Hospital, Southern Medical University, Guangzhou, 510515, China;[4] Department of Oncology-Pathology, Karolinska Institutet, Stockholm, Sweden;[5] Department of Cell and Molecular Biology, Karolinska Institute, S-171 76, Sweden; [6] Key Laboratory, The Second Hospital of Shandong University, Jinan, 250000, China; [7] Department of Microbiology, Tumor and Cell Biology, Karolinska Institute, S-171 76, Sweden; [8] Department of Nephrology, Qilu hospital;[9] Department of Pharmacology, The Second Hospital of Shandong University, Jinan, 250000, China;

***Correspondence should be addressed to:**

Jing Wu, PhD, Department of Pharmacology, The Second Hospital of Shandong University, 247#, Beiyuan Road,Jinan, 250000,Shandong Province, P.R. China.

Tel: 0737803095

Email: wujing@sdu.edu.cn

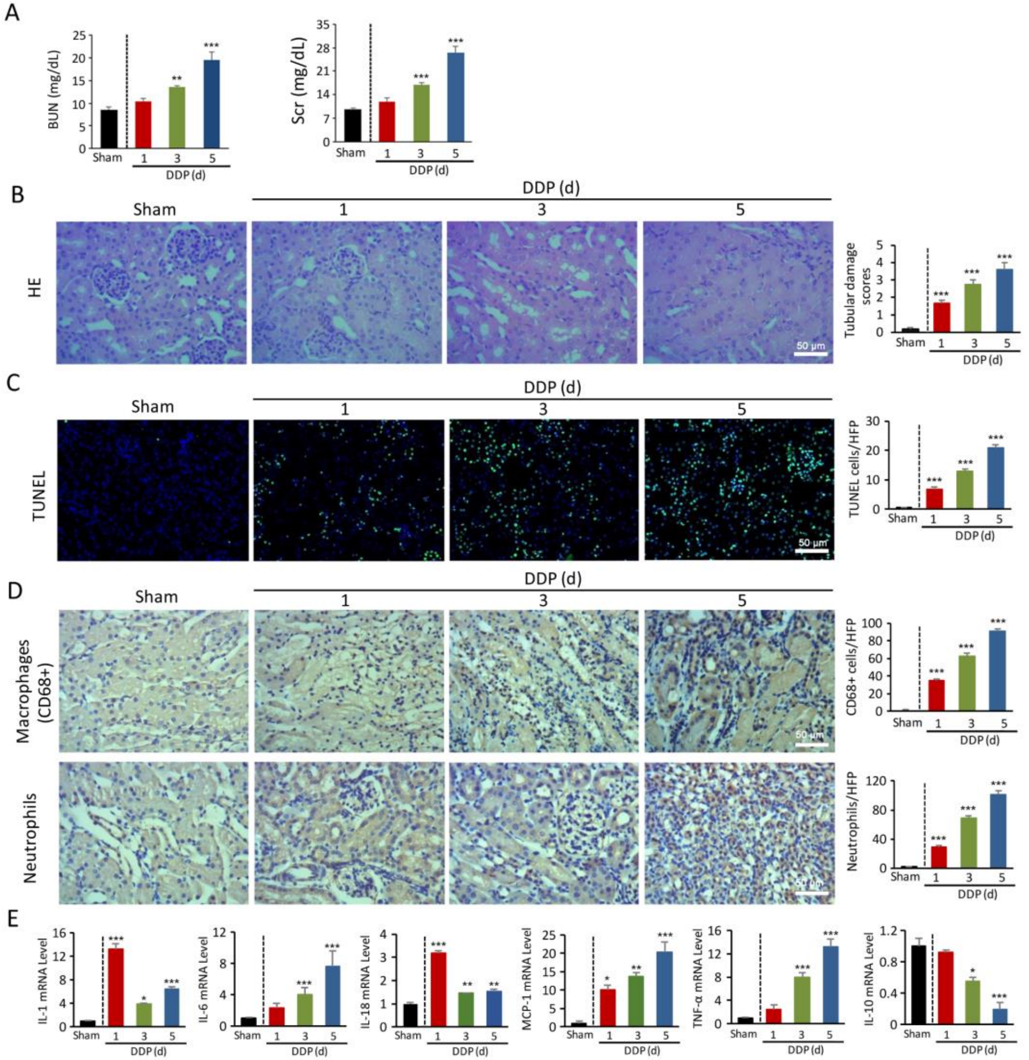
A
BUN (mg/dL)
Scr (mg/dL)
Sham
1
3
5
DDP (d)
B
HE
C
TUNEL
D
Macrophages (CD68+)
Neutrophils
Tubular damage scores
TUNEL cells/HFP
CD68+ cells/HFP
Neutrophils/HFP
50 μm
E
IL-1 mRNA Level
IL-6 mRNA Level
IL-18 mRNA Level
MCP-1 mRNA Level
TNF-α mRNA Level
IL-10 mRNA Level

Figure S1 Inflammation and apoptosis were increased dramatically in the kidney of DDPIN mice. The content of BUN and SCr (A) in the serum of DDPIN mice. HE staining (A and B) and TUNEL staining (C and D) in the kidneys of DDPIN mice. (E) Immunohistochemical staining of neutrophils and CD68+ macrophages and data analysis (F) in the kidneys of DDPIN mice. (G) RT-PCR analysis of IL-1, IL-6, IL-10, IL-18, MCP-1 and TNF-α. **P < 0.05, **P < 0.01, ***P < 0.001* versus control (n=6).

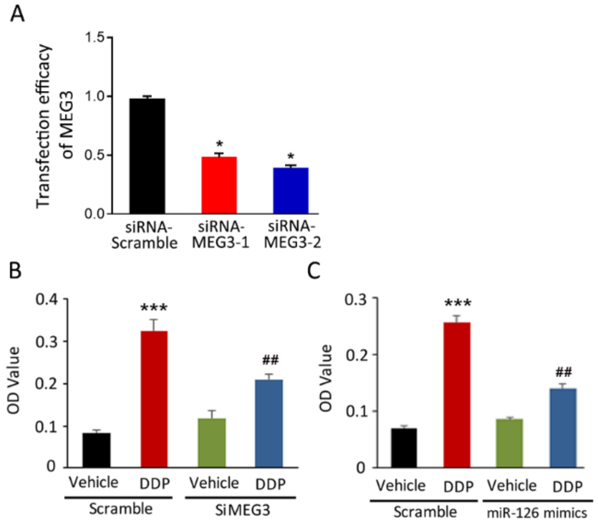

Figure S2 *lnc-MEG3* silence and miRNA-126 mimics reverse the apoptosis induced by DDP. (A)The transfection efficacy of siRNA-MEG3 were detected. MTT results of DDP-treated HK-2 cells in the condition of *lnc-MEG3* silence (B) and miRNA-126 mimics (C). **P < 0.05, **P < 0.01, ***P < 0.001* versus control, $^{\#}P < 0.05$, $^{\#\#}P < 0.01$, $^{\#\#\#}P < 0.001$ versus DDP-treated control (n=3).

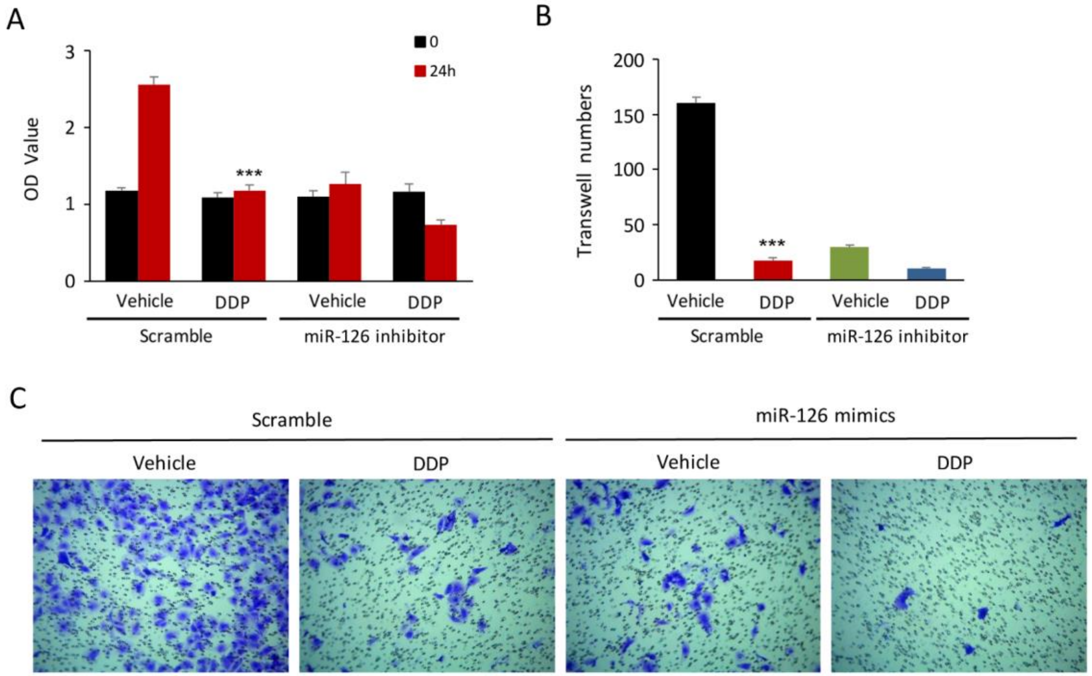

Figure S3 MiRNA-126 inhibitor aggravates the proliferation and migration of the DDP-treated HK-2 cells. (A) CCK8 assay, (B and C) Transwell assay of HK-2 cell and data analysis in the condition of miRNA-126 inhibitor. *$P < 0.05$, **$P < 0.01$, ***$P < 0.001$ versus control, # $P < 0.05$, # # $P < 0.01$, # # # $P < 0.001$ versus DDP-treated control (n=3).

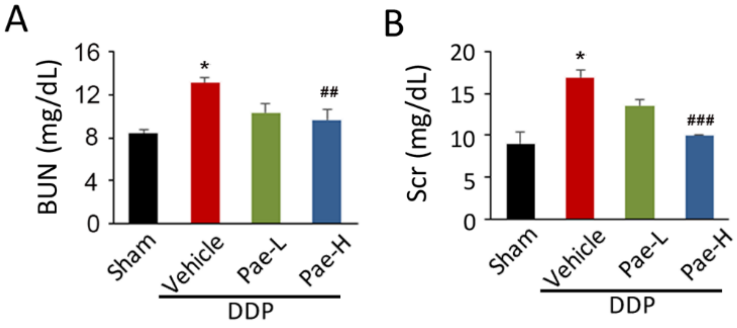

Figure S4 Pae improves the proliferation and migration of the DDP-treated HK-2 cells. (A) CCK8 assay, (B) Colony formation assay, Transwell assay of HK-2 cell (C) and data analysis. **P < 0.05, **P < 0.01, ***P < 0.001* versus control, $^{\#}P < 0.05$, $^{\#\#}P < 0.01$, $^{\#\#\#}P < 0.001$ versus DDP-treated control (n=3).

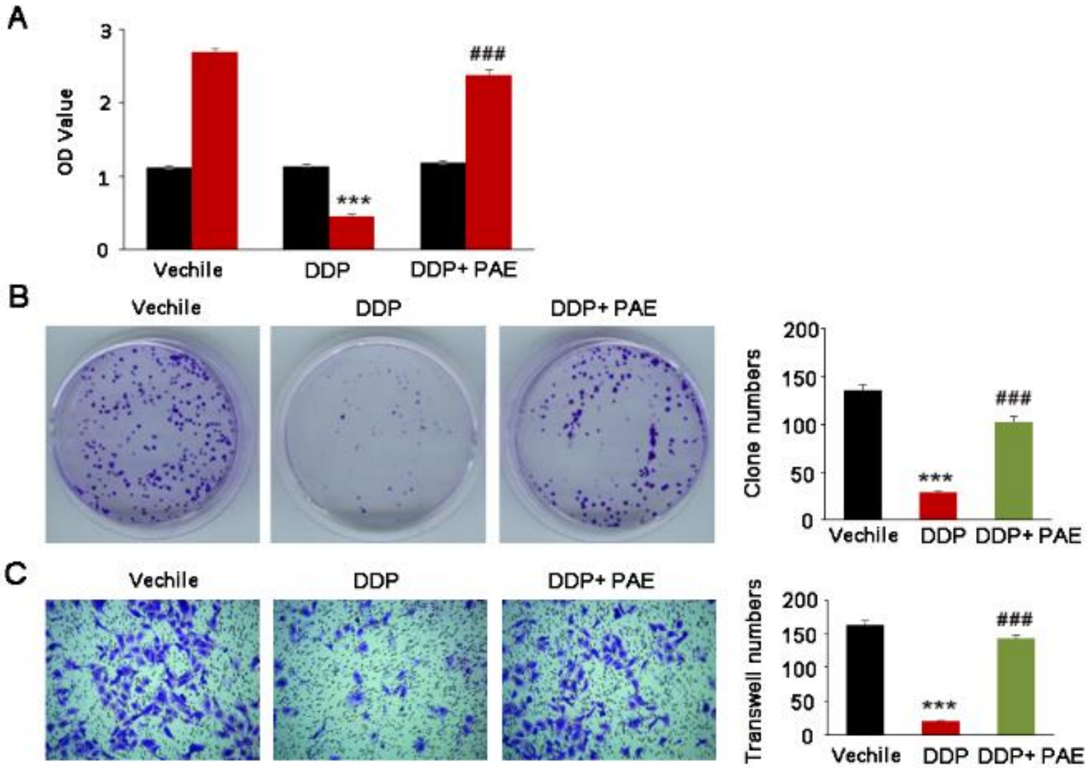

Figure S5 Pae affects the expression of *lnc-MEG3* and miRNA-126. Pae could downregulate the expression of *lnc-MEG3* (A)and upregulate the expression of miRNA-126 (B) in mice at the dose of 15 mg/kg and 30 mg/kg. $^{*}P < 0.05$, $^{**}P < 0.001$, $^{***}P < 0.0001$ versus control, $^{\#}P < 0.05$, $^{\#\#}P < 0.01$, $^{\#\#\#}P < 0.001$ versus DDP-treated control (n=3).

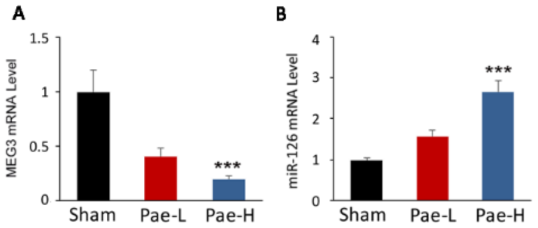

Figure S6 Pae reversed DDP-induced elevated BUN and SCr. Pae downregulates the content of BUN (A) and SCr (B) in the serum of DDPIN mice. *$*P < 0.05$, $**P < 0.001$, $***P < 0.0001$* versus control, $^{\#}P < 0.05$, $^{\#\#}P < 0.01$, $^{\#\#\#}P < 0.001$ versus DDP-treated control (n=3).

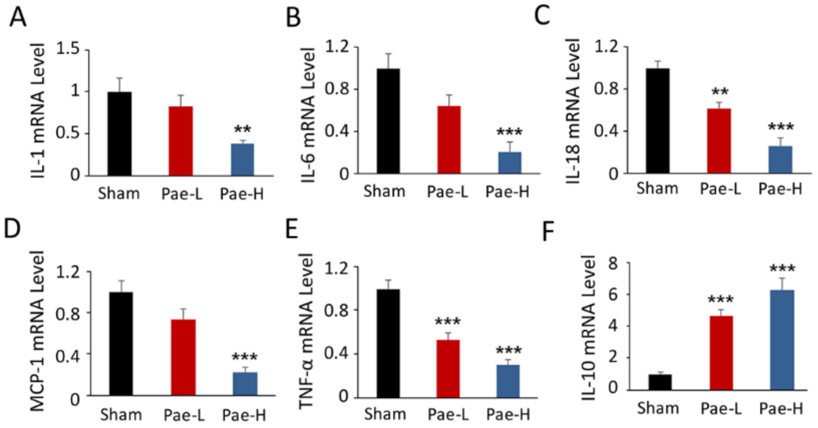

Figure S7 Pae influences inflammatory cytokines. Pae regulates the level of IL-1 (A), IL-6 (B), IL-10(C), IL-18(D), MCP-1(E) and TNF-α (F) in mice at the dose of 15 mg/kg and 30 mg/kg. *$*P < 0.05$, $**P < 0.01$, $***P < 0.001$* versus control (n=3).

Table S1. Antibodies used in this study

Primary antibodies	Host	Dilution and supplier	Catalog number	Application
AQP1	Goat	1:100; Santa Cruz, Dallas, TX	sc-9878	IF
Calbindin D28K	Mouse	1:100; Santa Cruz, Dallas, TX	sc-365360	IF
AQP3	Goat	1:100; Santa Cruz, Dallas, TX	sc-9885	IF
Neutrophil	Mouse	1:100; AbD Serotec, Oxford, UK	MCA805GA	IF
CD68	Mouse	1:100; AbD Serotec, Oxford, UK	MCA341GA	IF
LC3	Rabbit	1:500; Abcam, Cambridge, MA	ab51520	WB, IHC
TSC2	Rabbit	1:500; ProteinTech Group, Chicago, IL	24601-1-AP	WB, IHC
TSC1	Rabbit	1:500; ProteinTech Group, Chicago, IL	20988-1-AP	WB
AKT	Rabbit	1:500; ProteinTech Group, Chicago, IL	10176-2-AP	WB
p-AKT	Mouse	1:1000; ProteinTech Group, Chicago, IL	66444-1-Ig	WB

mTOR	Rabbit	1:1000; ProteinTech Group, Chicago, IL	20657-1-AP	WB
p-mTOR	Rabbit	1:500; Abcam, Cambridge, MA	ab109268	WB
TBC1D7	Mouse	1:1000; Santa Cruz, Dallas, TX	sc-514595	WB
Atg5	Rabbit	1:5000; ProteinTech Group, Chicago, IL	10181-2-AP	WB
Beclin-1	Rabbit	1:1000; ProteinTech Group, Chicago, IL	11306-1-AP	WB
GAPDH	Rabbit	1:1000; ProteinTech Group, Chicago, IL	10494-1-AP	WB

Table S2. Primer pairs of target genes used for real time RT-PCR in this study

Genes	Accession No.	Forward	Reverse
Mus TNF-α	NM_001278601.1	GAAAAGCAAGCAGCCAACCA	CGGATCATGCTTTCTGTGCTC
Mus IL-1β	NM_008361.4	CTGCAGCTGGAGAGTGTGG	GGGGAACTCTGCAGACTCAA
Mus IL-6	NM_031168.1	AGTTGCCTTCTTGGGACTGA	TCCACGATTTCCCAGAGAAC
Mus MCP-1	NM_011333.3	ACCTGCTGCTACTCATTCAC	TTGAGGTGGTTGTGGAAAAG
Mus IL-18	NM_001357221.1	ACTGGCTGTGACCCTCTCTG	TGGATCCATTTCCTCAAAGG
MEG3	NM_144513	TCCTCACCTCCAATTTCCCCT	GAGCGAGAGCCGTTCGATG
Mus β-actin	NM_007393.3	GGCTGTATTCCCCTCCATCG	CCAGTTGGTAACAATGCCATGT
MiRNA-126	NR_029713.1	GGA ATG TAA GGA AGT GTG	GAG CAG GCT GGA GAA
